# Supplementary material for: Structure and chemistry of graphene oxide in liquid water from first principles
Source: Nat Commun. 2020 Mar 26;11:1566. doi: 10.1038/s41467-020-15381-y (PMC7099009; doi:10.1038/s41467-020-15381-y)
Supplement: Supplementary file 1 — Supplementary Information [file 41467_2020_15381_MOESM1_ESM.pdf]

Supplementary Information for “Structure and Chemistry of Graphene Oxide in Liquid Water from First Principles”

Félix Mouhat, François-Xavier Coudert, and Marie-Laure Bocquet

CONTENTS

I. Supplementary Figures 3

II. Supplementary Tables 14

III. Supplementary Methods 16

III.1. Cutoff convergence tests 16

III.2. Simulation cell 17

III.3. Simulation movies 17

IV. Supplementary Discussion 18

IV.1. Dynamics of empty GO replicas 18

IV.2. Dehydration enthalpy 19

IV.3. Special epoxides 19

IV.4. Competition between intramolecular and intermolecular H bonds 20

IV.5. Stability of the GO in liquid water 20

IV.6. Influence of initial configuration 20

IV.7. Reactivity of random GO 20

IV.8. Chemical species/functions histograms 21

IV.9. H_2O Diffusion 22

Supplementary References 24

I. SUPPLEMENTARY FIGURES

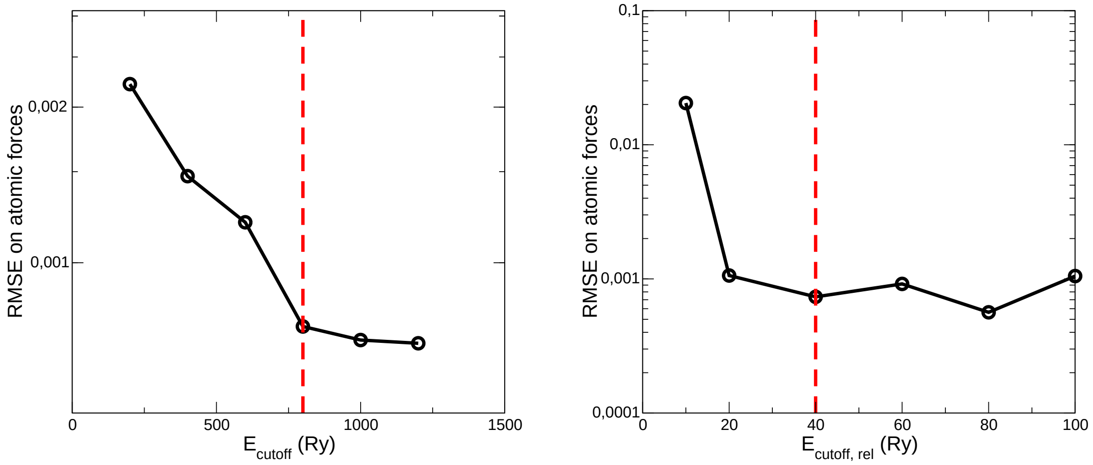

Supplementary Figure 1. Logarithmic Root Mean Square Error (RMSE) on atomic forces as a function of the main E_{cutoff} (left) and relative $E_{\text{cutoff, rel}}$ (right) cutoffs. Red dashed lines correspond to the selected values for the AIMD simulations discussed in the manuscript. All the tests presented here have been carried out with the MOLOPT basis set.

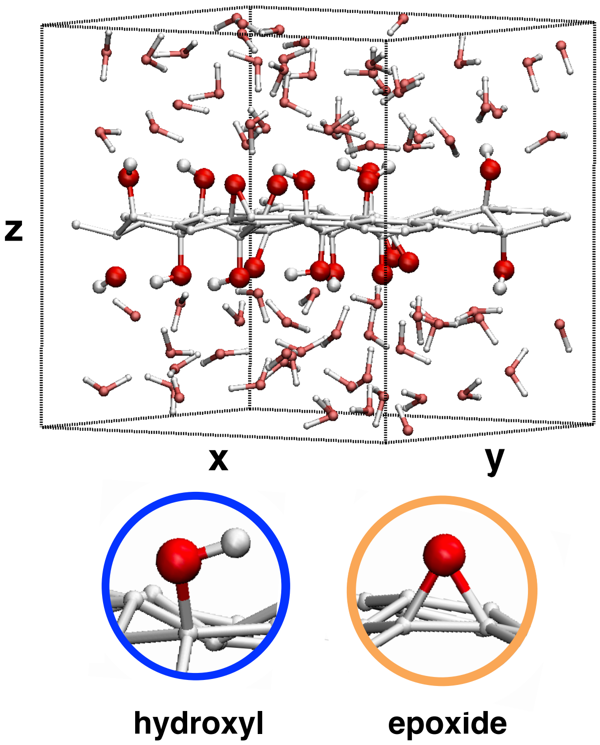

Supplementary Figure 2. VMD snapshot of the simulation cell. The GO containing 72 carbons, 18 oxygens and 12 hydrogens is placed in a $15 \times 13 \times 14.5$ Å^3 orthorhombic cell containing 80 H_2O molecules. Geometry and cell parameters optimization are then performed using the CP2K suite of codes.

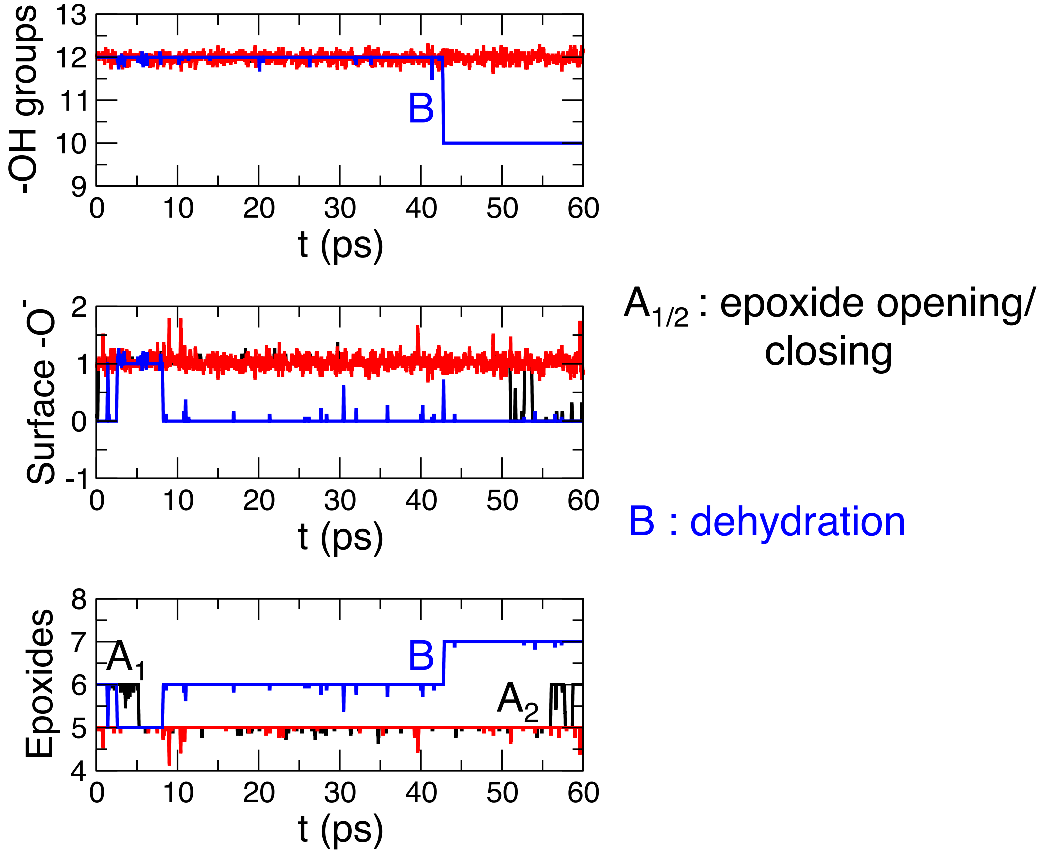

Supplementary Figure 3. Time evolution of the number of chemical functions present at the GO surface: hydroxyl groups (top), surface alcoholates (middle) and epoxides (bottom). The black, red and blue colors are associated with the semi-ordered 1, semi-ordered 3 and semi-ordered 4 GO replicas, respectively. At variance with simulations in liquid water, the reactive events for empty GO replicas are scarce.

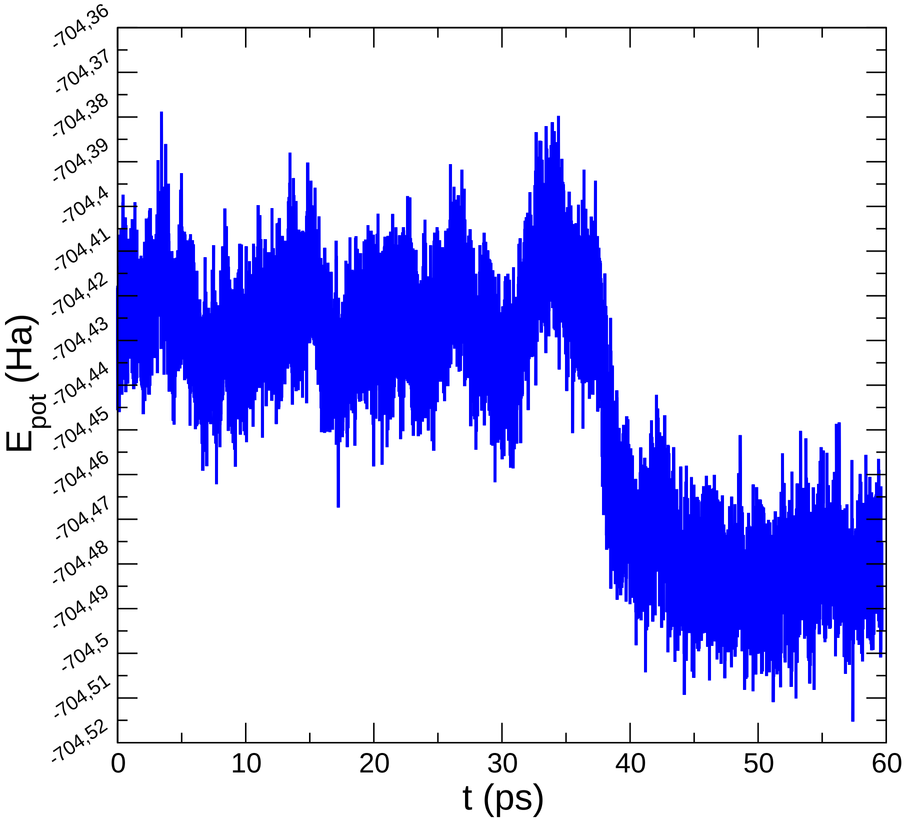

Supplementary Figure 4. Time evolution of the electronic potential energy E_{pot} along the AIMD trajectory of the empty semi-ordered 4 model at $T = 300$ K.

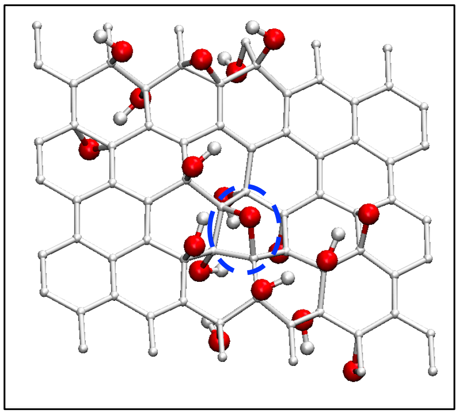

Supplementary Figure 5. 1,3-epoxide function in the replica semi-ordered 1. $\widehat{\mathrm{COC}} \sim 90°$instead of 60°, as reported in Figure **2c)**.

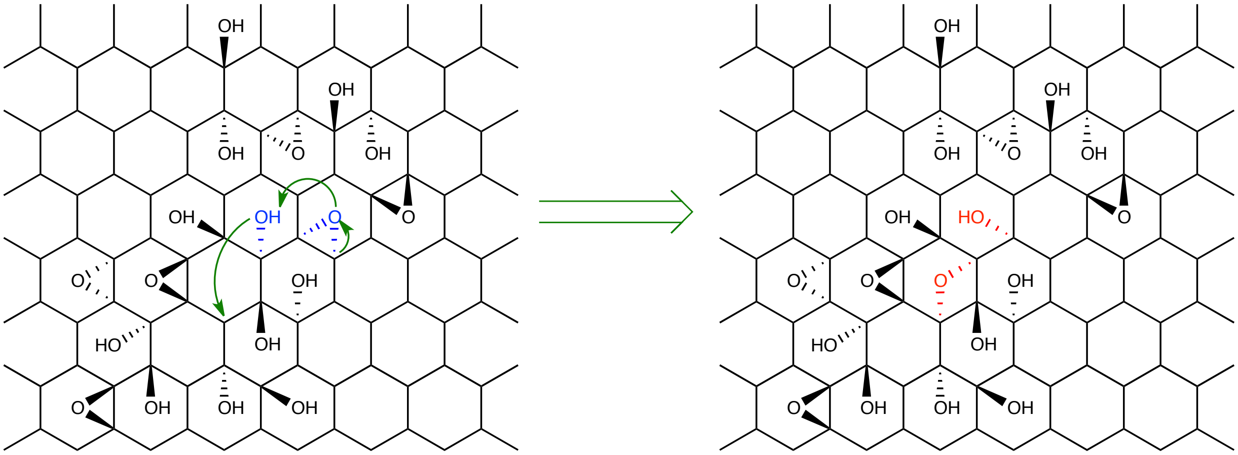

Supplementary Figure 6. Mechanism of formation of the 1,3-epoxide function in the replica semi-ordered 1. This electronic rearrangement occurs spontaneously at T = 0 K during the geometry optimization of the surface.

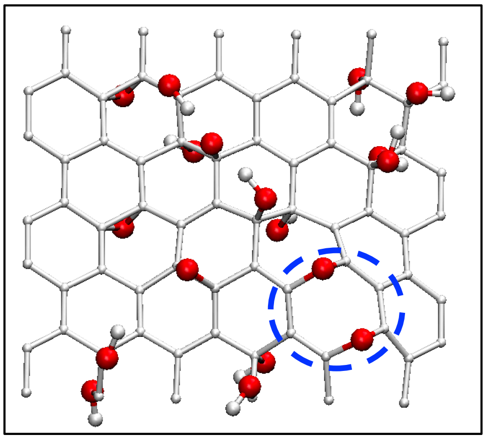

Supplementary Figure 7. Broken Bond (BB) epoxide functions in the replica random 4. $\widehat{\mathrm{COC}} \sim 105^\circ$ instead of 60°, as reported in Figure **2c)**.

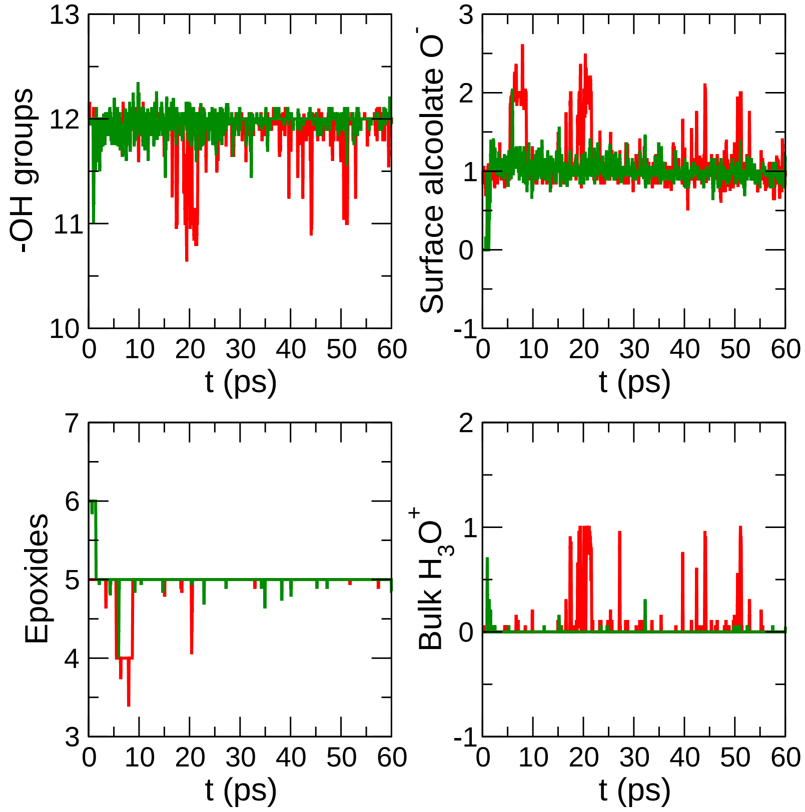

Supplementary Figure 8. Time evolution of the number of neutral functional groups of the GO model 3 (left) and charged species present at the surface or in the water bulk (right). The red and green colors are associated respectively to the first generation of water solvent and the second generation of water solvent.

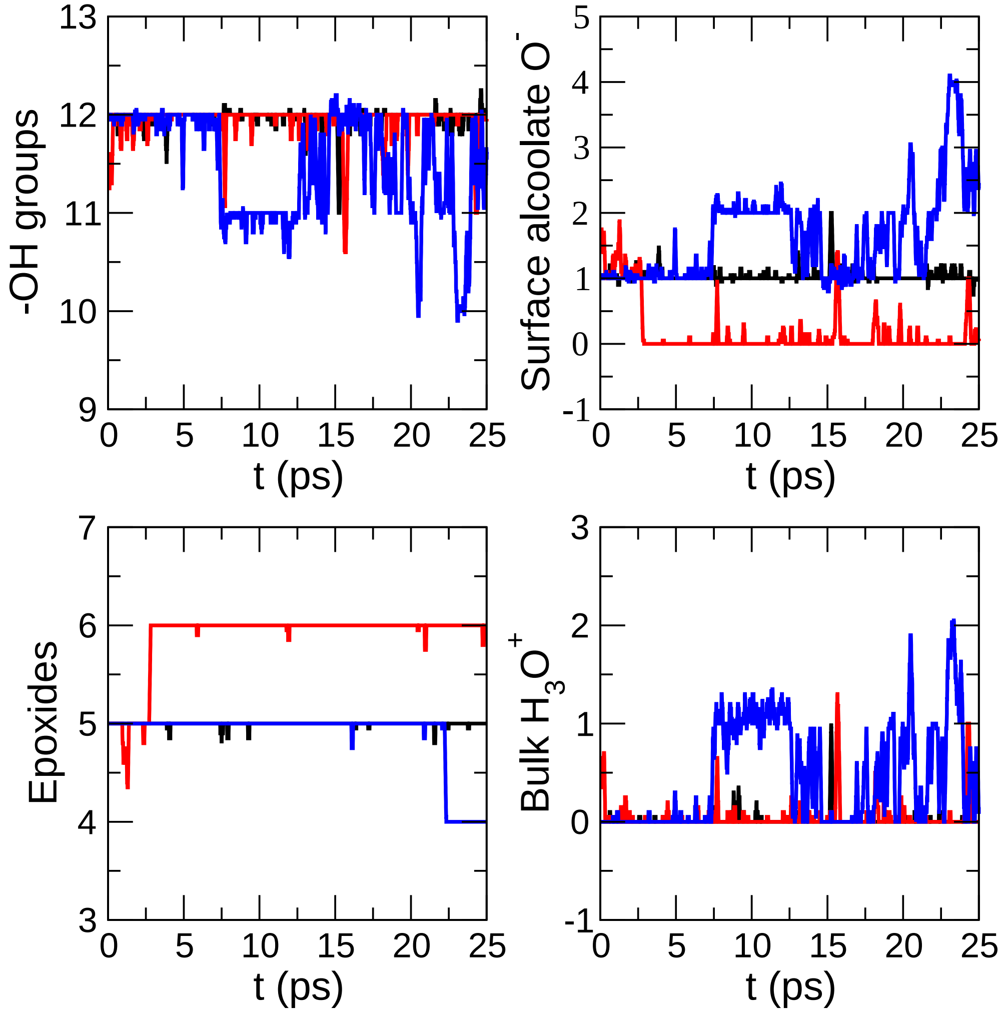

Supplementary Figure 9. Time evolution of the number of neutral functional groups of the GO surface (left) and charged species present at the GO surface or in the water bulk (right). The black, red and blue colors are associated to the random 1′, random 2′ and random 4′ GO models, respectively. The reactive events occurring along the random GO dynamics in water are similar to those discussed in the manuscript.

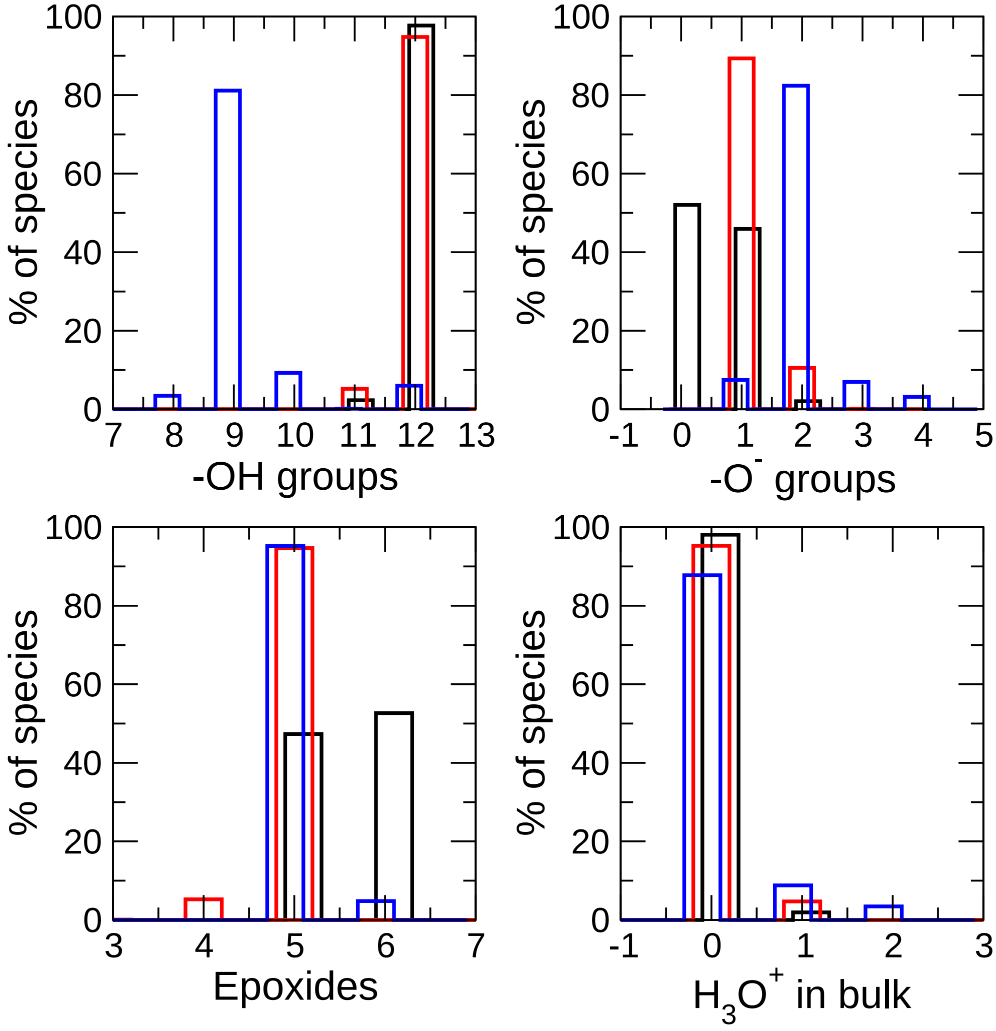

Supplementary Figure 10. Percentages of the neutral functional groups of the GO surface (left) and charged species present at the GO surface or in the water bulk (right). The black, red and blue colors are associated to the semi-ordered 1, semi-ordered 3 and semi-ordered 4 GO models, respectively.

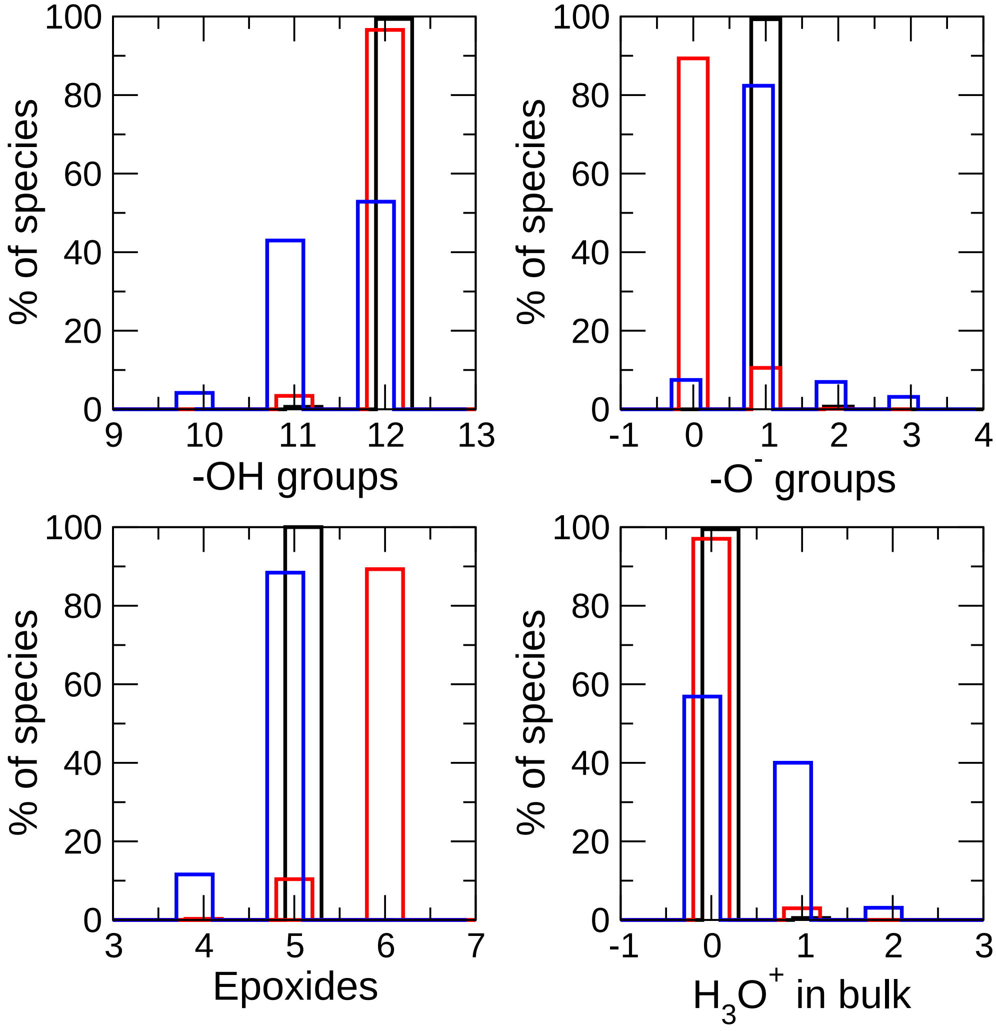

Supplementary Figure 11. Percentages of the neutral functional groups of the GO surface (left) and charged species present at the GO surface or in the water bulk (right). The black, red and blue colors are associated to the random 1′, random 2′ and random 4′ GO models, respectively.

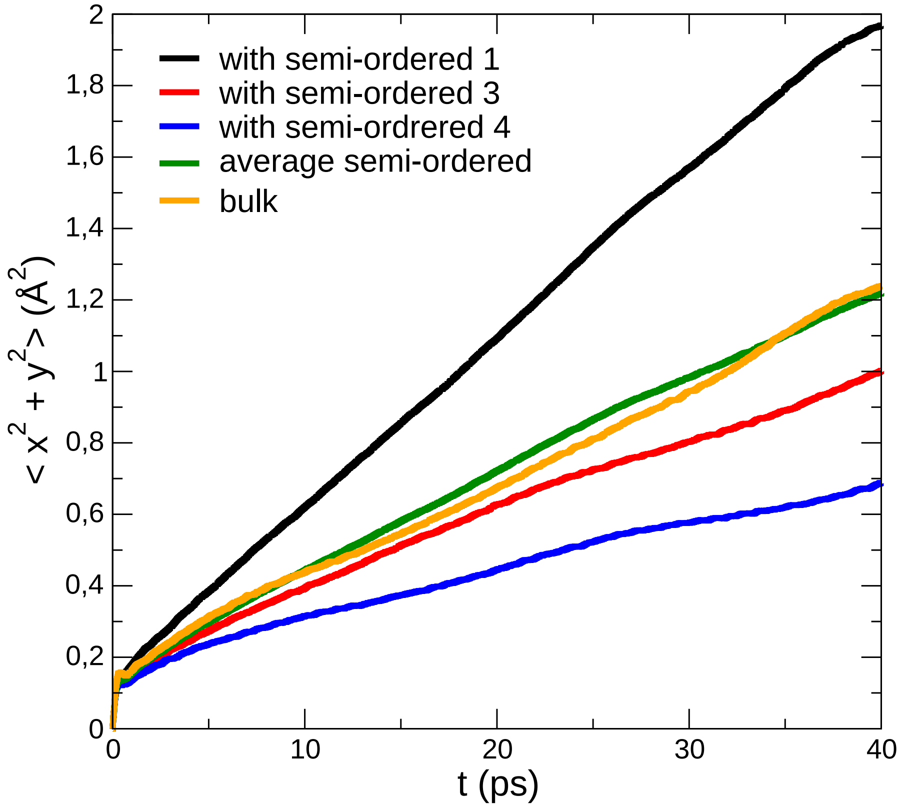

Supplementary Figure 12. Time evolution of the H_2O Mean Squared Displacement (MSD) (Eq. (2)) in the xy plane. The black, red and blue curve correspond to the diffusion of H_2O in presence of semi-ordered GO replicas while the bulk diffusion is plotted in green. The orange curve is obtained by averaging the H_2O diffusion over the 3 studied semi-ordered GOs.

II. SUPPLEMENTARY TABLES

GO configuration	empty material	in water
semi-ordered 1	5	3
semi-ordered 3	9	9
semi-ordered 4	6.5	4

Supplementary Table 1. Average number of intramolecular hydrogen bonds of the 3 most stable semi-ordered GO models from AIMD simulations, in vacuum and in liquid water.

GO configuration	E_{elec}	$\langle E_{\text{MD,empty}} \rangle$	$\langle E_{\text{MD,water}} \rangle$
semi-ordered 1	-704.50	-704.37	-2082.98
semi-ordered 3	-704.54	-704.46	-2083.03
semi-ordered 4	-704.53	-704.44	-2083.07
Average semi-ordered	-704.52	-704.42	-2083.03
random $1'$	-704.47	-704.34	-2082.92
random $2'$	-704.51	-704.36	-2082.93
random $4'$	-704.43	-704.29	-2082.89
Average random	-704.47	-704.33	-2082.91
Δ(Avg. SO – Avg. R) (eV)	-1.36	-2.45	-3.27

Supplementary Table 2. Energies of the 6 most stable GO replicas after various steps (structural relaxation, MD simulations of the empty and hydrated material) of the protocol detailed in the Methods section. All values are given in units of Hartree. Brackets indicate thermal averages performed over the AIMD trajectory.

GO configuration	Excess charge/GO surface unit (mC/m^2)
random 1′	−0.87
random 2′	−3.29
random 4′	−38.04
**Average**	**-14.06**

Supplementary Table 3. Excess charge in mC/m^2 on a random GO surface in contact with bulk water. See Table **4** for the result with semi-ordered GO surfaces.

Medium	$D_\parallel$
semi-ordered 1	1.12
semi-ordered 3	0.507
semi-ordered 4	0.281
Bulk (this work)	0.668
Bulk ([1])	1.8

Supplementary Table 4. Computed lateral diffusion coefficients $D_\parallel$ of H_2O in various media at room temperature (units:$\times 10^{-6}$ $cm^2.s^{-1}$).

III. SUPPLEMENTARY METHODS

III.1. Cutoff convergence tests

We detail below the procedure to determine the simulations parameters that are given in the Methods Section. The two main parameters that have been tested are the two cutoffs that need to be specified in the CP2K code [2], namely the "main" energy cutoff and the "relative" energy cutoff of the plane waves expansion. These cutoffs must be adapted according to the chemical system, the number of grids used by the Quickstep module during the DFT calculations ($N_{\text{grids}} = 4$, the default value), the functional (PBE-D3) and the basis set. Since we aim at performing *ab initio* Molecular Dynamics of the GO in water, a good convergence of the atomic forces is required. Therefore, we extracted one configuration from a short AIMD simulations and we evaluated the Born-Oppenheimer forces applying on each atom of the system, for various values of E_{cutoff} (which defines the planewave cutoff for the finest level of the multi-grid) and $E_{\text{cutoff, rel}}$ (which defines the planewave cutoff of a reference grid covered by a Gaussian with unit standard deviation). For each test, we compute the Root Mean Square Error (RMSE) on the forces, which is defined as

$$\text{RMSE} = \sqrt{\sum_{i=1}^{3N} (f_i - f_{i,0})^2} \tag{1}$$

with N the number of atoms of the system. f_i and $f_{i,0}$ are the Born-Oppenheimer forces evaluated at the tested cutoff and at a very large cutoff ($E_{\text{cutoff, max}} = 2500$ Ry and $E_{\text{cutoff, rel, max}} = 200$ Ry), respectively. The results of this convergence study are presented in Supplementary Figure **1**. The red dashed lines correspond to the values of E_{cutoff} and $E_{\text{cutoff, rel}}$ that we employed to perform all the calculations presented in this manuscript, corresponding to the best trade-off between accuracy and computational resources savings. We also ensured that the choice $E_{\text{cutoff}} = 800$ Ry and $E_{\text{cutoff, rel}} = 40$ Ry does not depend of the selected basis set for the DFT calculations. Indeed, the graphs obtained in Supplementary Figure **1** are similar when Double Zeta, Double Zeta for PBE, MOLOPT and MOLOPT_SR basis sets are tested. As mentioned in the manuscript and in the caption of Supplementary Figure **1**, we performed our calculations with the MOLOPT basis set.

III.2. Simulation cell

For each AIMD simulations presented throughout the manuscript and this Supplementary Information, the $15 \times 13 \times 14.5$ Å^3 orthorhombic simulation cell contains 80 H_2O molecules surrounding a semi-ordered or random GO replica. As explained in the manuscript, for the sake of simplicity, the GO surface only contains hydroxyls groups and epoxides functions, which are represented in Supplementary Figure **2**, under the simulation cell. Depending on the type of GO considered (semi-ordered or random), we generated 60 or 30 ps-long AIMD trajectories for production, after a 5 ps equilibration of the system.

III.3. Simulation movies

We provide two short movies generated using the Visual Molecular Dynamics (VMD) software with the Tachyon renderer [3, 4]. *Supplementary Movie 1* exhibits several proton exchanges between the surface hydroxyl groups and the surrounding H_2O molecules present in the bulk. This movie is extracted from the semi-ordered 3 trajectory but such processes are noticed in almost GO replicas, either random or semi-ordered. *Supplementary Movie 2* includes the more rare dehydration reaction that is observed only along the trajectory of the semi-ordered 4 GO in both vacuum and H_2O.

IV. SUPPLEMENTARY DISCUSSION

IV.1. Dynamics of empty GO replicas

To confirm the essential role played by the strong interaction between GO replicas and interfacial water molecules in the chemical reactivity of GO, we carried out AIMD simulations of the same semi-ordered GO replicas in the absence of water (in vacuum). For the sake of comparison, calculations were performed in exactly the same conditions (temperature, cell parameters, length of the trajectory) as those reported in the main text. We plot in the Supplementary Figure **3** the time evolution of the chemical functions present at the surface of each GO replica.

First, when comparing to Figure **5** of the main text, it is clear that very few reactive events occur during the empty GO dynamics. This constitutes a further indication of the stability and the reliability of the generated semi-ordered models. As expected, there is no deprotonation of the surface hydroxyl groups (see Supplementary Figure **3**, top panel), as in the bulk such events occur as a result from a strong hydrogen bonding with neighbouring H_2O molecules. We notice however, some opening/closing reactions of epoxide groups labelled as A_1 and A_2 in Supplementary Figure **3** (bottom panel). As discussed in the manuscript, these reactions correspond to an electronic rearrangement to a zwitterionic species, but do not create negative surface charge (unlike the deprotonation reactions). Finally, we observed in one GO model the rare dehydration reaction (labelled B): it occurs for the same semi-ordered 4 model as reported in the hydrated system in the manuscript. This reaction is due to the presence of a special motif in the structure: 3 consecutive hydroxyl groups with diaxial interactions on the same side of the basal carbon map. The three hydroxyl groups belong to two adjacent hexagons and are not subject to other intramolecular hydrogen-bonding with epoxides nearby. Therefore, two strong H bonds can be formed between these three groups and transformed to covalent bonds in the following. However, we notice that the dehydration reaction occurs in the empty GO dynamics much later than in the solvated system (after 40 ps of simulation instead of $\sim$ 5 ps in the presence of H_2O molecules), suggesting that the solvating H_2O molecules fasten the dehydration process.

IV.2. Dehydration enthalpy

We have followed the evolution of the potential energy of the system E_{pot} along the AIMD trajectory on the model 4 in vacuum in Supplementary Figure **4**. There is a clear step corresponding to the dehydration reaction. To estimate the enthalpy of this process, we performed averages of E_{pot} before and after the dehydration event, and evaluated the energy difference. Before dehydration we obtain $\langle E_{\text{pot,reac}}\rangle = -704.423$ Ha and after dehydration, $\langle E_{\text{pot,prod}}\rangle = -704.483$ Ha which corresponds to a dehydration enthalpy of $\Delta H = -0.05$ Ha. The dehydration enthalpy is therefore -131.3 kJ/mol.

IV.3. Special epoxides

We attempt here to interpret the presence of anomalous values of the $\widehat{\text{COC}}$ angle around 90° and 105° noticed in Figure **2c)** when the expected value for epoxides is around 60°. Indeed, epoxides can be classified into two groups: the commonly understood Total Triangle (TT) epoxides ($\widehat{\text{COC}} \sim 60°$) and the less frequent Broken Bond (BB) epoxides [5]. The BB epoxides, as indicated by their name, correspond to a C–O–C covalent bond where the chemical bond linking the two carbon sites has been broken, due to a too large C–C distance. Therefore, we visualized all the GO replicas studied throughout the manuscript and this SI and the BB epoxides we identified are spotted by a blue dashed circle in Supplementary Figures **5** and **7**.

In Supplementary Figure **5**, we observe a 1,3 epoxide in the semi-ordered 1 replica (such chemical functions are usually 1,2- in GO [6]), where the oxygen atom is bonded to two non-neighbours carbon atoms. Therefore, $\widehat{\text{COC}} \sim 90°$due to the larger distance between the involved carbon atoms.

We also found that this 1,3-epoxide is spontaneously created at T= 0 K during the geometry optimization, according to an electronic rearrangement pictured in Supplementary Figure **6**. During this transformation, we notice a simultaneous opening of a 1,2-epoxide whose oxygen captures a proton of a neighbour hydroxyl group. The oxygen of the hydroxyl group losing its proton, is unstable and closes back to a 1,3-epoxide because the first neighbouring carbons are already sp^3.

On the other hand, the value $\widehat{\text{COC}} = 105°$ stems from two epoxides that are located

in the same aromatic cycles, as shown in Supplementary Figure **7**. This highly substituted cycle is therefore certainly subject to an important mechanical strain. Consequently, the C–C bonds on which the two epoxides rely break to reduce the strain, forming two flat BB epoxides, explaining the $\widehat{\mathrm{COC}} = 10^\circ$ larger value.

IV.4. Competition between intramolecular and intermolecular H bonds

We have computed the number of intramolecular H bonds on every semi-ordered model in vacuum and in water. For model 1 and 4 the presence of water has broken 2 intramolecular bonds that are switching to intermolecular bonds. In contrast model 3 which is the most stable GO model in vacuum presents the same maximum number of intramolecular bonds both in vacuum and in water.

IV.5. Stability of the GO in liquid water

To complete the energetic analysis performed after geometry relaxation in vacuum, we have computed the average energy over the AIMD trajectories, first in vacuum and second in liquid water. Supplementary Table **2** provides the stability of semi-order models with respect to random models, in units of eV. The trend observed in vacuum is confirmed in liquid water: the semi-ordered models are more stable than random models.

IV.6. Influence of initial configuration

On the most stable model 3, we have checked the influence of the initial configuration of the water molecules. We compared two MD trajectories, starting from two different packings of water molecules, and the results are shown in Supplementary Figure **8**.

IV.7. Reactivity of random GO

In the GO models Section, we have evidenced the enhanced stability of the semi-ordered structures compared with the random ones. Therefore, we focused our discussion around the behaviour of anhydrous or wet semi-ordered GO. However, despite their structural differences, semi-ordered and random GOs should exhibit rather similar reactivity with the

surrounding H_2O molecules because the chemical functions at the surface are the same. As in Figure **5**, Supplementary Figure **9** displays the time evolution of neutral (hydroxyl and epoxide functions) and charged species (alcoolates and hydronium cations) present on GO and in bulk water for the three random models. First, the three replicas behave quite differently over time. The random $1'$ model (black) is almost inert, preserving a single alcoholate function at the surface, due to the partial opening of an epoxide function, where the excess positive is delocalized around this site. Similarly to semi-ordered models, we notice that all replicas start with 5 epoxides and not 6 showing that the initial geometry optimization process has resulted into one epoxide opening to certainly reduce the strain in the material. The random $4'$ GO (blue curve) appears to be the most reactive with more numerous proton or charge transfer occurring along the GO dynamics, more particularly around $t = 20$ ps. Finally, the random $2'$ replica (red curve) is also a little reactive exhibiting very few proton transfers and producing transient charging species.

We also evaluated the number of hydronium cations that will exactly compensate the GO charge (bottom right panel of Supplementary Figure **9**) and the resulting GO charges are shown in Supplementary Table **3** where average charges on GO are computed for the 3 random GO replicas. The negative charge values per replica are spread between 0.8 mC/m^2 to 40 mC/m^2 and yield the average value of 14 mC/m^2, which is slightly larger than in the semi-ordered case. However, larger trajectories of the random replicas are in principle needed to refine our conclusions although we expect this value to not significantly change.

IV.8. Chemical species/functions histograms

We started a chemical species analysis by plotting the evolution of the chemical species involved in the interaction between the GO surface and H_2O molecules in Figure **5** and in Supplementary Figure **9**. We therefore put in evidence that the reactivity of the different GO surfaces strongly depends on the surface itself which are more or less charged during the dynamics.

To complete this study, we represent the percentages of the different species present in the system in Supplementary Figures **10** and **11** for semi-ordered and random GOs, respectively. We confirm that the presence of H_3O^+ cations in the bulk is almost exclusively due to proton transfers ocurring between the surface hydroxyl groups and the surrounding

H_2O molecules that are H-bonded to them. In average, only 5-10 % of the hydroxyl groups are deprotonated and no more one single function is deprotonated at a given time of the GO dynamics. The epoxides functions, as discussed in the manuscript and in the previous paragraph, can dynamically close and open, creating an important surface charge. Indeed, disregarding the type of GO (semi-ordered or random), at least one epoxide function is opened and converted to a surface alcoolate during the major part of the GO dynamics. Such opening and closing processes do not directly induce spontaneous proton transfer between surface alcoolate and H_2O molecules in the bulk, and epoxides are in average stable despite their dynamical behaviour, justifying that they have been considered so far as metastable function at room temperature [7].

IV.9. H_2O Diffusion

We provide further investigations about the potential influence of the GO surface on the H_2O dynamics at room temperature. Indeed, we have evidenced in the manuscript and in the previous paragrah that a rich chemistry exists between the surface and the surrounding H_2 molecules. Therefore, it might impact the value of the self-diffusion constant D of H_2O molecules in this environment. In the following, we detail the procedure we employed to compute such diffusion coefficients. As mentioned in the manuscript, we have generated three 60 ps long AIMD trajectories of GO in water at $T = 300$ K, and a single trajectory of pure H_2O at $\rho = 0.99657$ g/cm^3. First, we removed the first 5 ps of each MD trajectory to disregard the equilibration period. Then, we applied a coordinates transformation to make the center of mass of the simulated system motionless during the MD trajectory. Afterwards, we computed the Mean Squared Displacement (MSD) in the xy plane, given by

$$\mathrm{MSD} = \langle |\mathbf{r}(t) - \mathbf{r}(0)|^2 \rangle_{xy} = \frac{1}{2N} \sum_{i=1}^{2N} (r_i(t) - r_i(0))^2, \tag{2}$$

where r_i are the atomic coordinates. The diffusion coefficient D can be estimated by calculating the slope of the MSD in the linear regime of t. Looking at Supplementary Figure **12**, we notice that the diffusion of water varies with the considered GO replica, depending on the chemistry occurring at the GO/water interface. However, this dependency is less important than with usual bidimensional surfaces and in average the diffusion of water in presence of GO is similar to the bulk diffusion. As it is known that the diffusion of water molecules

tends to be inhibited in confined media, this observation suggests that the dynamics of the H_2O molecules is presumably accelerated for smaller interlayer values. Such observations have already been reported in ref. [8], where the MD trajectories have been generated with the DREIDING force field to describe the GO surface.

Despite the shortness of our AIMD trajectories to compute accurate and reliable diffusion coefficients, we report in Supplementary Table **4** the values of the parallel (*i.e.* in the xy plane) H_2O self-diffusion coefficient D, computed by taking the slope of the MSD (see Eqn. 2). The computed diffusion coefficient D all have the same order of magnitude, albeit a bit smaller than the value reported in the literature, confirming the preliminary observation of Supplementary Figure **12**).

SUPPLEMENTARY REFERENCES

[1] Pham, T. A., Ogitsu, T., Lau, E. Y. & Schwegler, E. Structure and dynamics of aqueous solutions from pbe-based first-principles molecular dynamics simulations. *J. Chem. Phys.* **145**, 154501 (2016).

[2] Hutter, J., Iannuzzi, M., Schiffmann, F. & VandeVondele, J. Cp2k: atomistic simulations of condensed matter systems. *WIREs Comput. Mol. Sci.* **4**, 15–25 (2014).

[3] Humphrey, W., Dalke, A. & Schulten, K. VMD – Visual Molecular Dynamics. *J. Mol. Graph.* **14**, 33–38 (1996).

[4] Stone, J. An efficient library for parallel ray tracing and animation. *Master Thesis, Comput. Sci. Dep., Univ. of Missouri-Rolla* (1998).

[5] Hunt, A. *et al.* Epoxide speciation and functional group distribution in graphene oxide paper-like materials. *Adv. Funct. Mater.* **22**, 3950–3957 (2012).

[6] Lerf, A., He, H., Forster, M. & Klinowski, J. Structure of graphite oxide revisited. *J. Phys. Chem. B* **102**, 4477–4482 (1998).

[7] Kim, S. *et al.* Room-temperature metastability of multilayer graphene oxide films. *Nature Mater.* **11**, 544–549 (2012).

[8] Devanathan, R., Chase-Woods, D., Shin, Y. & Gotthold, D. W. Molecular dynamics simulations reveal that water diffusion between graphene oxide layers is slow. *Sci. Rep.* **6**, 457 (2016).
