# Supplementary material for: Structure and chemistry of graphene oxide in liquid water from first principles
Source: Nat Commun. 2020 Mar 26;11:1566. doi: 10.1038/s41467-020-15381-y (PMC7099009; doi:10.1038/s41467-020-15381-y)
Supplement: Supplementary file 3 — Description of Additional Supplementary Files [file 41467_2020_15381_MOESM3_ESM.pdf]

Description of Additional Supplementary Files

File name: Supplementary movie 1
Description: Movie showing several proton exchanges between the surface hydroxyl groups and the surrounding H2O molecules present in the bulk.

File name: Supplementary movie 2
Description: Movie showing the dehydration reaction that is observed in the trajectory of the semi-ordered 4 graphene oxide model in liquid water.
